# Graphitic carbon nitride for biomedical applications: challenges and future opportunities in dentistry

**DOI:** 10.1186/s11671-026-04650-2

**Published:** 2026-05-27

**Authors:** Manal Matoug-Elwerfelli, Swapna Thomas, Hadeel Kheraldine, Sara Adan Mahmoud, Asad Dabbour Asad, Abdalrahman Alkahlout, Ahmed Abdou, Hani Nazzal, Monty Duggal, Aboubakr M. Abdullah Ali

**Affiliations:** 1 College of Dental Medicine, QU Health, Qatar University, Doha, Qatar; 2 College of Medicine, QU Health, Qatar University, Doha, Qatar; 3 Independent Researcher, Doha, Qatar; 4 Department of Restorative Dentistry, Faculty of Dentistry, University of Malaya, Kuala Lumpur, Malaysia; 5 Paediatric Dentistry, Hamad Medical Corporation, Doha, Qatar; 6 Center for Advanced Materials, Qatar University, Doha, Qatar; 7 Faculty of Dentistry, Kings College London, London, United Kingdom

**Keywords:** Graphitic carbon nitride, G-C_3_N_4_, Synthesis, Composition, Physicochemical properties, Drug delivery, Wound healing, Biomedical, Dental

## Abstract

Graphitic carbon nitride (g-C_3_N_4_) is an emerging nanomaterial with unique features such as photocatalyst, biocompatibility, and tunable surface chemistry, placing g-C₃N₄ as a versatile platform for innovative biomedical applications. This review explores the properties, synthesis, biomedical applications, challenges, and future perspectives of g-C₃N₄. An electronic search of PubMed and Elsevier’s Scopus was undertaken, with no limits to the year of publication, and only English-language literature was included. One of the notable applications is as a drug delivery system due to its large surface area, functional tunability, and controlled release mechanisms. Recently, g-C_3_N_4_ has also emerged as a promising agent for cancer treatment, particularly in photodynamic and photothermal therapies. Another widely explored application is its role in regenerative medicine, including wound healing, where its biocompatibility and ability to enhance cellular interactions are advantageous. Additionally, g-C_3_N_4_ demonstrates strong potential in biosensing and bioimaging, leveraging its fluorescence properties for diagnostic and monitoring applications. While direct studies in dentistry are limited, these biomedical advancements suggest a potential for future dental applications, particularly in antimicrobial coatings, drug-loaded restorative materials, and guided tissue regeneration. Future research should focus on optimizing g-C_3_N_4_-based materials for targeted clinical applications, particularly within dentistry.

## Introduction

The pursuit of metal-free catalytic materials has led to extensive research into carbon-based alternatives, among which graphitic carbon nitride (g-C₃N₄) has emerged as a promising candidate due to its semiconducting polymeric structure and tunable physicochemical properties [1]. The discovery of carbon nitride dates back to 1834, when Liebig identified melamine, melem, melam, and 'melon', which are considered fundamental building units of triazine and tri-s-triazine frameworks [2]. Although initially overlooked, subsequent analysis by Franklin [3] and Pauling & Sturdivant [4] clarified its structure and bonding, laying the foundation for modern g-C₃N₄ in chemistry. A major breakthrough occurred in 2009, when Wang et al. demonstrated visible-light-driven hydrogen production using g-C₃N₄, establishing it as an efficient metal-free photocatalyst [1]. Since then, research has expanded rapidly, exploring its synthesis, modification, and application across photocatalysis, energy conversion, and biomedicine.

Structurally, g-C₃N₄ consists of triazine units interconnected through nitrogen linkages, forming a multi-layered two-dimensional (2D) framework that provides remarkable robustness and tunable surface chemistry [1, 5, 6]. Its adjustable bandgap, chemical and thermal stability, intrinsic antimicrobial activity, and low-cost synthesis position it as a promising candidate for biomedical and dental applications [7–10]. One of the most compelling features of g-C₃N₄ is its versatile biomedical potential, particularly in drug delivery, where its large surface area and weak intermolecular interactions facilitate drug loading and controlled release for therapeutic agents such as anticancer drugs [11–14]. Additionally, g-C₃N₄ demonstrates photocatalytic and bioactive properties, enabling applications in photodynamic (PDT) and photothermal therapy (PTT) [1, 15].

The bioactivity of g-C₃N₄ further supports tissue interactions, wound healing, and tissue regeneration [16, 17]. Silver-embedded g-C₃N₄ has demonstrated potent antifungal effects against *Candida albicans*, a prevalent oral pathogen, by disrupting fungal membrane and biofilm formation, with minimum inhibitory concentrations (MIC) ranging from 16 to 256 µg/mL, while maintaining excellent biocompatibility with fibroblastic cells up to 1000 µg/mL [18]. Furthermore, its electrical and fluorescent properties facilitate biosensing and bioimaging, enabling real-time monitoring of biological processes [19–22]. These properties open avenues for diagnostic applications, including the detection of biomolecules, biomarkers, and pathogens, an area of growing interest in precision medicine [23–25].

Despite its demonstrated biomedical promise, the application of g-C₃N₄ within dentistry remains largely unexplored. Conventional dental materials often lack antimicrobial resilience, bioactivity, and long-term functional stability. Advances in dental materials increasingly rely on bioactive compounds that promote tissue regeneration and enhance clinical outcomes [26, 27]. Exploring g-C₃N₄-based nanomaterials for dental adhesives, restorative materials, implant coatings, and regenerative therapies could address several unmet clinical needs. This review provides an integrative and translational perspective, it consolidates recent advances in drug delivery, cancer therapy, tissue engineering, wound healing, and biosensing within a precision biomedical framework, highlighting stimuli-responsive and imaging-guided applications. Furthermore, it extends beyond conventional biomedical discussions to systematically explore the clinical relevance and translational trajectory of g-C₃N₄ for dentistry, an area underrepresented in the literature. Moreover, it critically examines key challenges, including synthesis scalability, reproducibility, dispensability, and in vivo safety, thereby identifying practical barriers and future research directions. By integrating multidisciplinary evidence and highlighting unmet clinical needs, this review advances the current understanding of g-C₃N₄ nanomaterials beyond existing reports.

## Literature search and scope of the review

An electronic literature search was conducted in PubMed and Elsevier’s Scopus to identify relevant studies on g-C₃N₄ and its biomedical and dental applications. The search strategy included combinations of keywords and MeSH terms such as “graphitic carbon nitride”, “polymeric carbon nitride”, “g-C₃N₄”, “biomedical applications”, “tissue engineering”, “wound healing”, “cancer therapy”, “bioimaging”, “biosensing”, “photodynamic therapy”, “photothermal therapy”, “drug delivery,” and “dental materials” using appropriate Boolean operators (AND/OR). The search was performed without publication year restrictions and was limited to English-language articles.

All retrieved records were screened at the title and abstract level independently by two authors (S.T and S.M). Any disagreements regarding eligibility were resolved by consultation with a third author (M.M.E). Reference lists of included studies were also manually screened to identify additional relevant publications. Given the broad interdisciplinary scope of g-C₃N₄ research, this review adopts an integrative narrative approach, focusing on thematic synthesis rather than systematic quantitative aggregation. Studies were selected based on relevance to material synthesis, physicochemical properties, biological interactions, therapeutic applications, and translational potential, with particular emphasis on emerging dental applications.

## Structural and physiochemical properties of g-C₃N₄

**Fig. 1:**
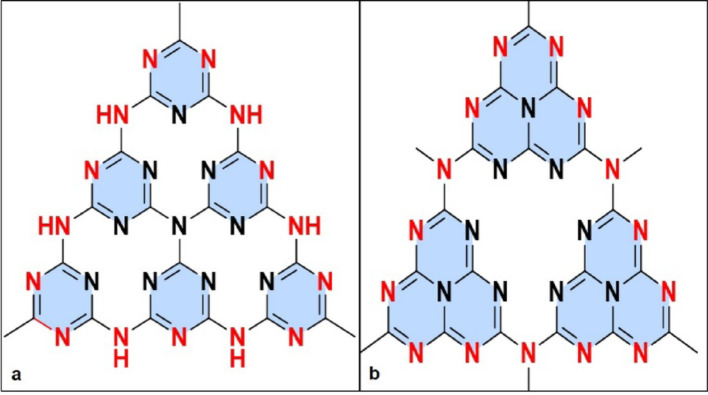
Illustration of g-C_3_N_4_ structural frameworks. **a** triazine and **b** heptazine (tri-s-triazine)

**Fig. 2:**
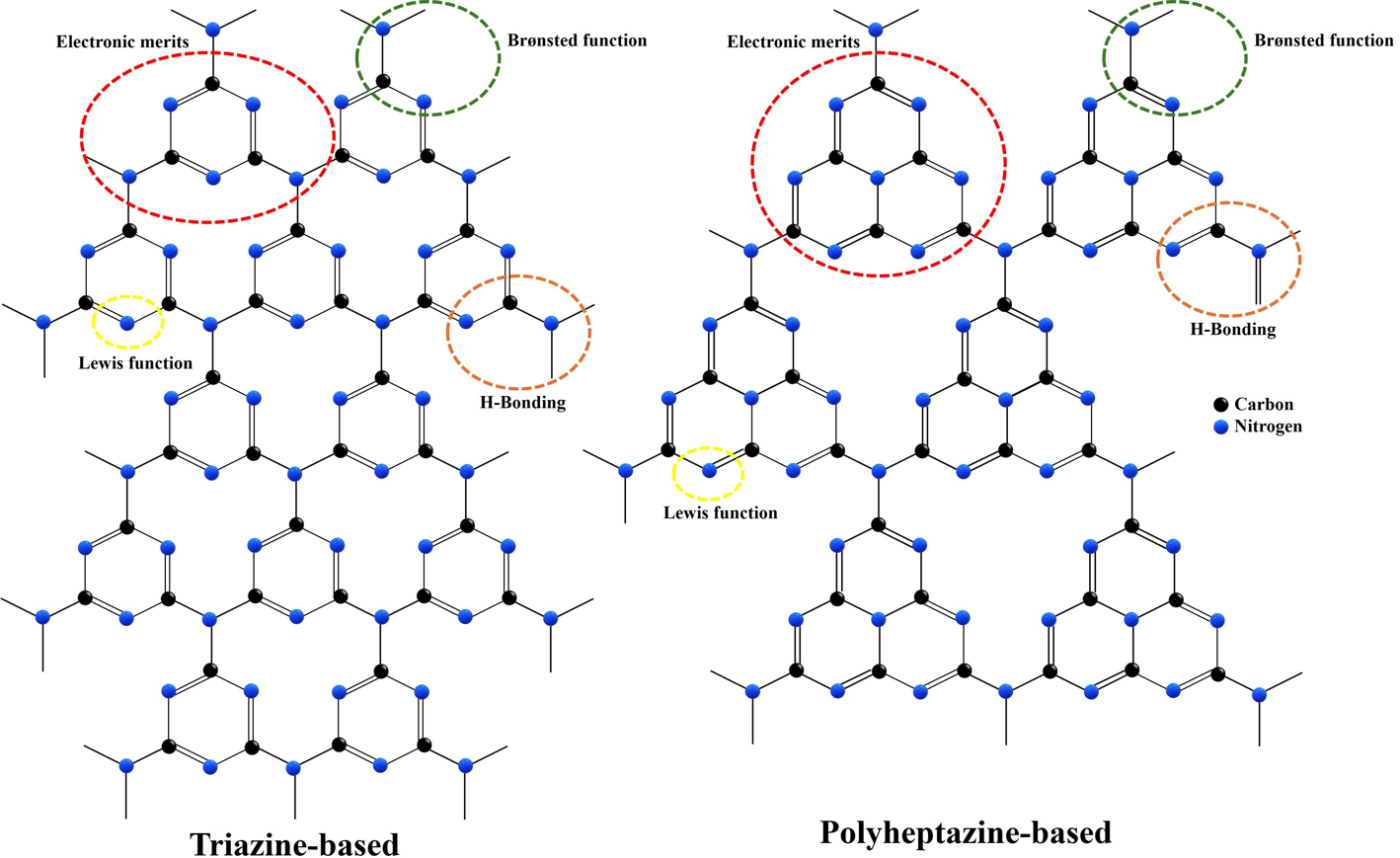
Molecular structure and surface properties of g-C_3_N_4_. The illustration highlights the triazine- and polyheptazine-based structures and their key surface functionalities. The material’s electronic merits are foundational for its conductivity in biosensing applications. The Brønsted and Lewis functions, along with hydrogen-bonding (H-bonding) motifs, serve as active sites for surface functionalization, which is critical for targeted drug delivery and enhancing interactions with biological systems

g-C₃N₄ is a polymeric, metal-free semiconductor composed exclusively of carbon and nitrogen atoms arranged in triazine or heptazine (tri-s-triazine) frameworks (Fig. 1), representing a thermodynamically stable carbon nitride allotrope [1]. The layered structure resembles graphite but features nitrogen substitution within the carbon network, conferring exceptional thermal stability up to 600 °C and chemical resilience across wide pH ranges [6, 28]. From a structural perspective, s-triazine (C₃N₃) and tri-s-triazine (C₆N₇) units polymerize into extended 2D sheets characterized by an indefinite number of layers [29]. Additionally, the material’s architecture incorporates abundant surface-active sites, including Lewis-base functionalities, Brønsted basicity, hydrogen-bonding motifs, and electron-rich regions [6, 28], as illustrated in Fig. 2. The above-mentioned properties are known to enhance its adsorption capacity and facilitate charge transfer processes, essential for photocatalytic and biomedical performance [30].

In terms of electronic structure and bandgap engineering, g-C₃N₄ functions as an n-type crystalline semiconductor with promising applications in photocatalysis. Its electronic band structure arises from the integration of carbon and nitrogen atoms with 2.7 eV bandgaps [31]. Theoretical computations have elucidated that the valence band of g-C_3_N_4_ is constituted by the 2p orbitals of nitrogen, whereas the conduction band is collectively formed through the overlap of the 2p orbitals belonging to carbon and nitrogen atoms [32]. Crucially, the highest occupied molecular orbital (HOMO) is mainly derived from all nitrogen entities; whereas the least occupied molecular orbital (LUMO) is broadly allocated among all carbon atoms in combination with nitrogen atoms. Consequently, during the process of photocatalysis, the nitrogen centre functions as an oxidation site, whereas all carbon atoms and nitrogen atoms serve as reduction sites.

**Fig. 3:**
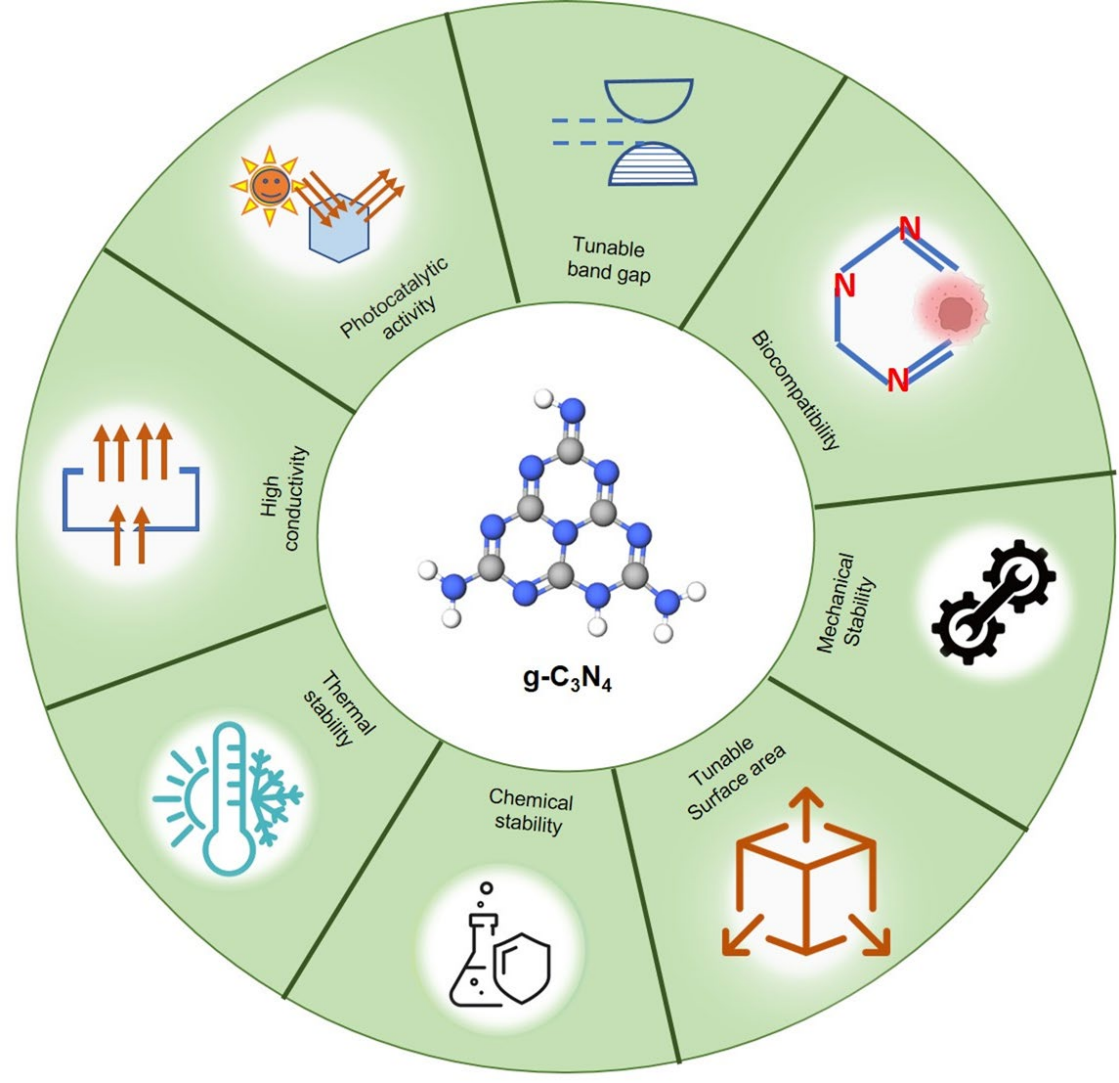
Schematic illustration of physical, chemical and mechanical properties of g-C₃N₄ that are specifically pertaining to biomedical applications. Each property directly impacts clinical performance such as; photocatalytic activity: Enables the generation of reactive oxygen species for photodynamic cancer therapy and antimicrobial treatments. Tunable band gap: Allows for optimization for specific applications like bioimaging and light-activated therapies. Biocompatibility: Essential for safe in vivo use, preventing adverse immune responses. Mechanical stability: Crucial for the durability of load-bearing dental and orthopedic implants. Tunable surface area: Allows for high drug loading capacity in delivery systems and enhanced sensitivity in biosensors. Chemical and Thermal stability: Ensures material integrity and longevity in the physiological environment. High conductivity: Underpins its use in developing sensitive electronic biosensors for diagnostics

Furthermore, bandgap engineering through non-metal doping or transition metals which modulates electronic properties, facilitates charge separation, and enhances carrier mobility [1, 5], Morphologically, g-C₃N₄ can be synthesized through different techniques into various forms, such as bulk, nanosheets, and mesoporous structures, each exhibiting distinct properties [33]. The structural properties of g-C₃N₄ can be further tailored through porosity modulation, surface engineering, and the incorporation of dopants or co-catalysts, which enhance its surface area and charge migration efficiency [34–36]. Bandgap engineering in g-C₃N₄ has been reported to correlate with enhanced photocatalytic and biomedical performance, particularly in reactive oxygen species (ROS) generation. Pristine g-C₃N₄ exhibits a bandgap of ~ 2.7 eV, which limits visible-light utilization and results in moderate ROS yield. However, bandgap narrowing to ~ 2.2–2.5 eV via non-metal (e.g., sulphur and phosphorus) or transition metal doping has been shown to enhance light absorption and suppress electron–hole recombination. This modification has been associated with reported increases in ROS generation (e.g., singlet oxygen and superoxide radicals) of up to approximately 2–5 fold, leading to improved antibacterial efficacy and photodynamic therapeutic outcomes [37]. Quantitative indicators such as reduced photoluminescence intensity and increased photocurrent response further support improved charge separation, which is closely linked to enhanced ROS-mediated biological activity in cancer and microbial models [38]. Collectively, these findings indicate a measurable relationship between bandgap tuning and photocatalytic/biomedical performance, supporting the role of engineered g-C₃N₄ in phototherapeutic applications. Such tunability underpins its versatility for bioimaging, drug delivery, and phototherapy, where electronic structure directly influences biological interactions. Figure 3 illustrates the most commonly reported g-C₃N₄ physical, chemical and mechanical properties of specific interest within the biomedical field.

## Synthesis strategies of g-C₃N₄

**Fig. 4:**
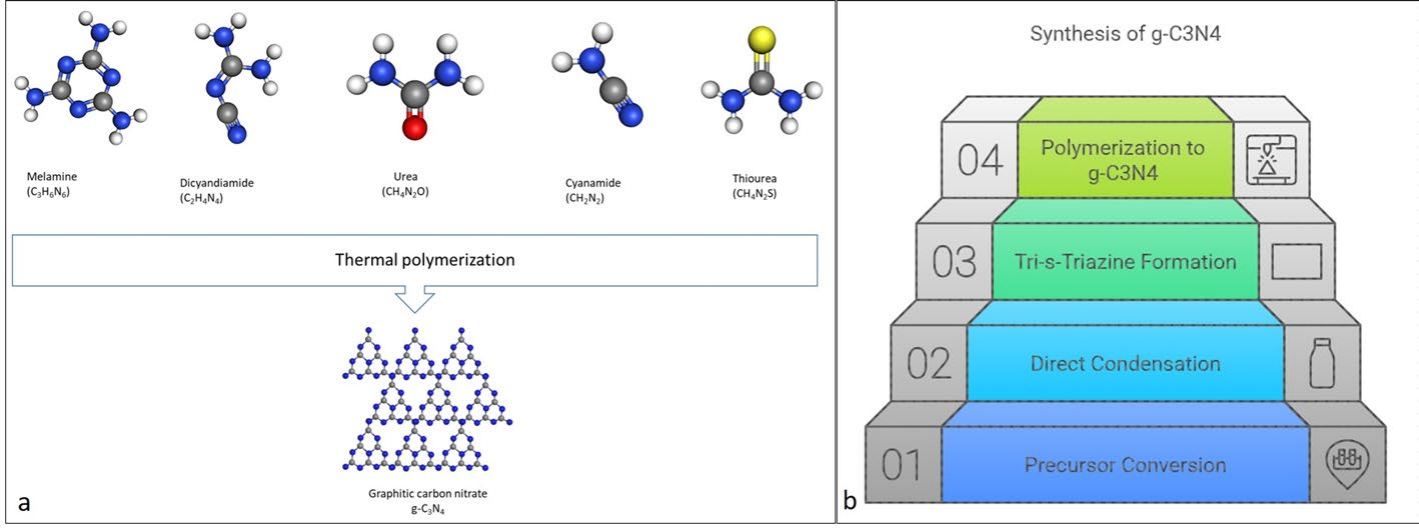
**a** Illustrating synthesis pathway of g-C_3_N_4_. **b** Most common precursors synthesized via thermal polymerization pathway

The synthesis of g-C₃N₄ is primarily based on the thermal polymerization of nitrogen-rich precursors (e.g., dicyandiamide, cyanamide, melamine, or urea) [1, 5], as illustrated in Fig. 4. Typically conducted at 450–550 °C, the process involves sequential nucleophilic addition and polycondensation reactions, during which intermediates such as melamine and melem condense into tri-s-triazine units to form the final graphitic framework [1, 5].

The physicochemical properties of g-C₃N₄, including morphology, crystallinity, defect density, and surface area, are strongly governed by precursor chemistry, heating rate, temperature profile, and reaction atmosphere, which collectively influence optical and electronic performance. To enhance functionality, post-synthetic strategies such as non-metal doping (e.g., boron, fluorine, phosphorus, sulfur) and surface functionalization are frequently employed to modulate band structure and interfacial behavior [6, 39].

Beyond conventional pyrolysis, advanced approaches including template-assisted and microwave-assisted synthesis have been developed to achieve greater control over nanostructure and porosity, thereby expanding the material’s potential for biomedical and dental applications.

### Pyrolysis and thermal polymerization

Pyrolysis remains the most widely adopted and scalable method for synthesizing g-C₃N₄. It involves controlled thermal condensation of nitrogen-rich precursors, producing layered polymeric carbon nitride structures [40]. The reaction typically proceeds through thermal decomposition, intermediate formation (melamine/melam/melem), and subsequent condensation into tri-s-triazine frameworks [41].

Within the current literature, there is a general consensus that the physicochemical characteristics and photocatalytic behaviour of g-C₃N₄ are strongly dictated by synthesis parameters, including precursor type, heating rate, temperature profile, and reaction atmosphere. Comparative studies, such as that of Ismael et al. [42], demonstrated that the utilization of various nitrogen-rich precursors produces g-C₃N₄ with distinct morphologies and porosities even under identical calcination conditions [42]. Such evidence highlights the sensitivity of g-C₃N₄ formation to subtle changes in precursor chemistry or synthesis environment, reinforcing the importance of precise thermal control for optimizing its functional properties.

Furthermore, recent studies have refined the pyrolysis approach to enhance the yield, surface area, and electronic performance of g-C₃N₄. For instance, Mou et al. [43] developed a one-step pyrolysis route using a deep eutectic solvent system (ZrOCl₂·8 H₂O and urea), producing g-C₃N₄/ZrO₂ lamellar composites with superior photocatalytic activity [43]. Similarly, Zhang et al. [44] synthesized highly porous g-C₃N₄ nanotubes through the pyrolysis of supramolecular melamine–cyanuric acid assemblies, resulting in enhanced visible-light absorption [44]. Mechanistic investigations by Mukhopadhyay et al. [45] further revealed parallel condensation pathways involving ammelide and cyanuric acid intermediates, providing new insights into structural evolution during thermal polymerization [45]. More recently, Lei et al. [46] introduced a wood-assisted pyrolysis–etching strategy that generated carbon vacancies without compromising structural integrity, thereby improving photocatalytic performance [46]. Although simple and industrially scalable, pyrolysis offers limited precision in morphological control, motivating the development of alternative synthesis strategies.

### Template-assisted synthesis

Template-assisted synthesis of g-C₃N₄ has emerged as a versatile strategy to tailor morphology, porosity, and surface chemistry; key parameters that determine its photocatalytic and biomedical performance. This method employs hard or soft templates to control the nanostructure of g-C₃N₄ during polymerization, allowing precise structural manipulation [47, 48]. For example, metal chloride salts such as calcium chloride (CaCl₂), potassium chloride (KCl), and sodium chloride (NaCl) function as sacrificial templates to improve crystallinity and textural features [49]. Meanwhile, biological templates, including apricot and mushroom biomass, have been successfully employed to synthesise manganese oxide composites (MnO₂–g-C₃N₄/C), where g-C₃N₄ enhances reactive sites and electrical conductivity [50]. Thus compared with bulk pyrolysis, template-mediated synthesis enables superior textural engineering, facilitating the development of nanostructured g-C₃N₄ systems with improved bioactivity and potential biomedical functionality.

### Microwave assisted synthesis

Microwave-assisted synthesis has emerged as a rapid and energy-efficient alternative, offering shorter reaction times and improved structural uniformity compared with conventional thermal polymerization [51]. Uniform volumetric heating accelerates polymerization and facilitates formation of nanoarchitectures such as nanosheets and nanorods with increased surface area [52]. This approach has been widely employed to fabricate functional composites. Chen et al. [53] synthesized mesoporous 3D g-C₃N₄ via a microwave-assisted hydrothermal route with enhanced visible-light absorption and improved CO₂ reduction efficiency [53]. Matias et al. [54] produced g-C₃N₄/TiO₂ heterostructures by combining microwave treatment with calcination, resulting in improved photocatalytic activity [54].

Similarly, Saxena et al. [55] fabricated g-C₃N₄/molybdenum disulfide (MoS₂) nanocomposites using a microwave-assisted process that yielded superior microstructural uniformity and increased surface area [55]. Venkatesh et al. [56] also reported a one-step microwave-assisted approach for synthesizing g-C₃N₄/copper oxide composites with an increased surface area and photocatalytic performance [56]. More recently, Li et al. [57] demonstrated the rapid synthesis of potassium and phosphorus co-doped g-C₃N₄, significantly improving H₂O₂ production and light absorption capacity [57]. Overall, microwave-assisted synthesis offers improved reproducibility and nanoscale control, attributes particularly valuable for biomedical applications.

### Comparative perspective on synthesis methods

Each of the above synthesis strategy offers distinct advantages. Pyrolysis provides scalability and simplicity but limited structural precision. Template-assisted methods enable controlled porosity and enhanced surface engineering, albeit with increased complexity. Microwave-assisted routes offer rapid processing and improved nanoscale uniformity. In the context of advancing carbon-based nanomaterials for biomedical applications, synthesis strategies play a critical role in determining their functional performance and translational potential. Recent studies comparing bottom-up and top-down synthesis routes highlight a key trade-off between structural control and scalability. Bottom-up approaches offer superior tunability and optical properties but face challenges in reproducibility and large-scale production, whereas top-down methods are more scalable yet often yield heterogeneous structures [58, 59]. Recent studies further highlight this trade-off between structural control and scalability in carbon-based nanomaterial synthesis [60]. Future progress may lie in hybridizing these approaches to balance scalability with structural control, thereby optimizing g-C₃N₄ nanomaterials for future dental translation (Table 1).

**Table 1:** Comparative overview of major g-C₃N₄ synthesis strategies and their respective advantages, limitations, and relevance to biomedical and dental applications

Synthesis method	Key features	Advantages	Limitations	Relevance to biomedical/ dental applications
Pyrolysis/ thermal polymerization	High-temperature (450–550 °C) condensation of nitrogen-rich precursors (e.g., urea, melamine, dicyandiamide)	Simple, low-cost, scalable; well-established; suitable for bulk production	Limited control over morphology and porosity; lower surface area; higher defect density; batch-to-batch variability	Suitable for large-scale fabrication of antimicrobial fillers, bulk coatings, and composite dental materials
Template-assisted synthesis	Use of hard or soft templates (salts, biomaterials, supramolecular assemblies) to guide nanostructure formation	Precise control over porosity, surface area, and morphology; improved charge separation; enhanced bioactivity	Multistep processes; template removal required; increased complexity and cost	Ideal for regenerative scaffolds, implant coatings, and systems requiring high surface reactivity
Microwave-assisted synthesis	Rapid, uniform heating via microwave irradiation, often combined with hydrothermal or doping strategies	Short reaction times; energy-efficient; high reproducibility; fine control over particle size and doping	Limited scalability; specialized equipment; optimization required for uniformity	Promising for precision biomedical and dental applications requiring reproducibility, nanoscale control, and functional tuning

## Biomedical applications of g-C_3_N_4_

**Fig. 5:**
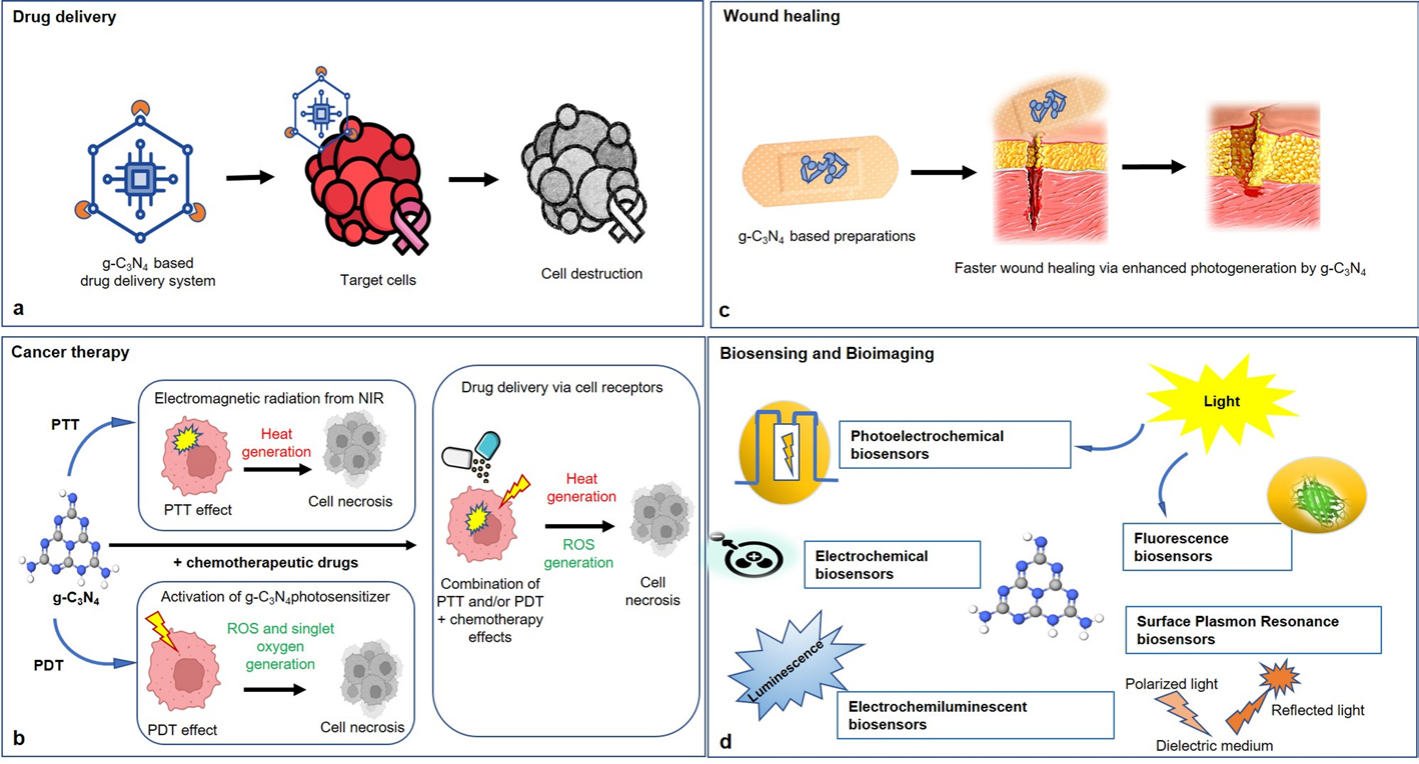
Different applications of g-C_3_N_4_**-**based materials in the biomedical field. **a** Drug delivery. **b** Cancer therapy. **c** Wound healing. **d** Biosensors and bioimaging

Taking into consideration the versatile and promising properties of g-C_3_N_4,_ namely its antimicrobial activity, biocompatibility, and potential for photothermal and photodynamic therapy, various g-C₃N₄-based nanocomposite materials have attracted significant research interest in the medical field. The most reported biomedical applications are illustrated in Fig. 5 and briefly discussed below.

### Drug delivery

The potential of g-C₃N₄ as a versatile drug delivery system (DDS) is one of its most promising biomedical applications, with several recent studies summarized in Table 2. The material’s large, π-conjugated surface area and tunable chemistry facilitate the efficient loading of various therapeutic agents, from conventional chemotherapeutics (doxorubicin and cisplatin) to natural compounds such as curcumin and quercetin, primarily through non-covalent interactions like hydrogen bonding and π-π stacking [13]. Using density functional theory calculations on various anticancer drugs, Perveen et al., reported that cisplatin can be stably adsorbed onto g-C₃N₄ via non-covalent interactions, highlighting g-C₃N₄ promise as a drug carrier while underscoring the need for experimental validation of release behaviour and in vivo performance [12]. Similarly, melphalan also exhibited a stable yet reversible non-covalent binding to nitrogen sites on g-C₃N₄, indicating its suitability as a nanocarrier for controlled anticancer drug delivery [14].

A key advantage highlighted across numerous studies is the ability to engineer platforms for controlled, stimuli-responsive release. This strategy allows drug offloading to be triggered by specific internal or external cues. For example, systems have been designed to leverage the acidic tumor microenvironment for pH-sensitive release, while others use external light irradiation to enable spatiotemporal control over drug delivery [14, 61, 62]. Furthermore, the performance and targeting of these nanocarriers can be significantly enhanced through strategic functionalization and composite formation. Ligand-based targeting, such as conjugating g-C₃N₄ nanosheets with folic acid, has proven effective for receptor-mediated delivery to specific cancer cells [63–65].

Despite the growing number of g-C₃N₄-based drug delivery systems reported in the literature, critical differences exist in drug loading capacity, release kinetics, and translational maturity [12, 66]. Drug loading efficiency is primarily governed by the surface area, porosity, and surface functional groups of g-C₃N₄, with non-covalent interactions such as π-π stacking, hydrogen bonding, and electrostatic attraction playing a dominant role [11]. Indeed, g-C₃N₄ conjugated π-electron system and nitrogen-rich surface enable strong non-covalent interactions with aromatic and heterocyclic drugs, facilitating high loading efficiency without the need for covalent modification [67]. Furthermore, strategies incorporating surface functionalization or composite formation (e.g., with polymers, metal oxides, or metal-organic frameworks) consistently demonstrate improved loading capacities and enhanced stability compared with pristine g-C₃N₄ [68]. Release kinetics reported across studies are predominantly stimulus-responsive rather than passive, with pH-, light-, and redox-triggered mechanisms enabling controlled and site-specific drug release [7, 61, 69]. While such responsiveness is advantageous for targeting pathological microenvironments, variations in synthesis method and surface modification result in heterogeneous release profiles, limiting direct comparison between systems [70].

Importantly, the majority of studies remain confined to in vitro models, often under simplified conditions that do not fully recapitulate physiological complexity. Comprehensive pharmacokinetic, biodistribution, and long-term toxicity assessments remain scarce. These gaps highlight the need for systematic in vivo studies and standardized evaluation metrics before g-C₃N₄-based DDS can be realistically advanced toward clinical translation.

**Table 2:** Summary of most recent in vitro applications of g-C_3_N_4_-based nanomaterials as a drug delivery system (DDS)

Drug delivery system (DDS)	Anticancer drug	Target tissue/cells	Assessment methods	Findings	Refernces
Zeolitic-imidazolate framework-8/Doxorubicin (g-C_3_N_4_/ZIF-8/DOX)	Doxorubicin	A549	Cell cytotoxicity assay, singlet oxygen detection, dual-colour fluorescence imaging	pH-responsive drug release, dual-colour fluorescence imaging, and imaging-guided photochemo therapy, good biocompatibility, and efficient singlet oxygen generation	[71]
g-C_3_N_4_ /Doxorubicin	Doxorubicin	Lipid bilayer membranes	Molecular dynamics simulations with POPC and cholesterol	Strong hydrogen bonding and p-p interaction, enhanced van der Waals and electrostatic energy contributions between g-C_3_N_4_ and Doxorubicin, facilitating rapid membrane binding and drug delivery	[11]
Chitosan/Agarose/g-C_3_N_4_/curcumin (CS/Ag/g-C_3_N_4_/Cur)	Curcumin	MCF-7	Cell cytotoxicity assay	High loading and entrapment efficiency of curcumine significantly decreased cancer cell viability (~ 12%) compared with free curcumin	[61, 62]
Halloysite/Chitosan/g-C_3_N_4_/Quercetin (HNT/CS/g-C_3_N_4_/QC)	Quercetin	MCF-7	Cell cytotoxicity assay	Good stability of DDS and effective release at pH 5.4–7.4, improved cytotoxicity compared to free Quercetin	[72]
Folic acid/g-C_3_N_4_/silver sulfide (FA/g-C_3_N_4_/Ag_2_S)	Folic acid	MCF-7	Cell cytotoxicity assay, photothermal therapy	Improved cytotoxicity due to the relatively high photothermal heating effect on MCF-7 cells	[64]
PLA/PVA/CS/FU/g-C_3_N_4_/DOX/PTX	Doxorubicin, 5-FU and paclitaxel	MCF-7	Cell viability assay	High cell cytotocixity due to controlled drug delivery and anti-tumar activity of tri-layer nanofibers	[73]

DDS, Drug delivery system; A549, human lung carcinoma epithelial cell line; MCF-7, human breast adenocarcinoma cell line

### Cancer therapy

In recent years, g-C_3_N_4_ has also emerged as a promising nanomaterial for cancer treatment, with several recent studies summarized in Table 3. Beyond simple compositional modifications, recent investigations have focused on engineering electronic band structures and surface chemistry to optimize light absorption, charge separation efficiency, and ROS yield. Coupling g-C₃N₄ with semiconductor or metallic nanoparticles (e.g., Zinc oxide and copper-based systems) has been shown to improve photoabsorption and suppress electron–hole recombination, thereby enhancing phototherapeutic performance [74]. The intrinsic photocatalytic activity of g-C₃N₄ under visible-light irradiation underpins its application in both PTT and PDT [69, 75]. Upon irradiation, g-C₃N₄ can convert photon energy into heat, inducing localized hyperthermia and subsequent tumour cell damage [13, 76]. Simultaneously, photoexcitation promotes ROS generation, leading to oxidative stress-mediated apoptosis through membrane disruption, mitochondrial dysfunction, and DNA damage [77, 78]. However, the magnitude of these effects remains highly dependent on irradiation parameters, tumour depth, and nanoparticle dispersion, factors that are not consistently standardized across studies.

Surface functionalization with targeting ligands such as antibodies, peptides, or carbon-based nanocarriers has been employed to enhance tumour accumulation and improve therapeutic precision [76, 79]. Although these modifications improve cellular uptake and tumour localization in preclinical models, concerns remain regarding immunogenicity, off-target distribution, and insufficient long-term biodistribution data, which may hinder clinical translation. In addition, near-infrared (NIR) activation has been explored to overcome the limited tissue penetration of visible light [13, 76]. However, the intrinsic NIR absorption of pristine g-C₃N₄ is relatively weak, frequently requiring integration with secondary photothermal agents, thereby increasing structural complexity and potentially affecting safety and reproducibility. Importantly, the majority of the current photodynamic and photothermal studies involving g-C₃N₄ are conducted using superficial tumor models under controlled irradiation conditions. The penetration depth of visible and NIR light under clinically relevant fluence rates remains limited, and robust validation in deep-tissue or clinically representative tumor settings is still lacking. An emerging strategy involves combining PDT with chemotherapy or chemodynamic therapy to achieve synergistic tumour suppression [80]. These multimodal systems enable concurrent ROS generation and localized hyperthermia, improving cytotoxicity compared with monotherapies. Nevertheless, the individual contribution of each therapeutic component is often insufficiently quantified, limiting mechanistic insight and rational optimization of these hybrid platforms.

A notable advantage of g-C₃N₄ is its photocatalytic water-splitting capability, enabling oxygen generation under light irradiation. This feature may alleviate tumour hypoxia, a major barrier to effective PDT [65, 81]. However, whether the oxygen generated in situ is sufficient to meaningfully overcome hypoxic gradients within solid tumours remains to be rigorously validated under clinically relevant conditions. Similarly, incorporation of catalytic components such as MnO₂ or copper-based species aims to exploit endogenous H₂O₂ within the tumour microenvironment [82, 83]. Li et al. reported a multifunctional nanoplatform based on g-C₃N₄ loaded with MnO₂ and CuS that not only induces PDT/PTT but generates oxygen in the tumor microenvironment to alleviate hypoxia, improving anticancer effects [82]. Additionally, g-C₃N₄ nanostructures coated with polydopamine (PDA) have demonstrated dual functionality, serving not only as a potent PDT agent under visible light but also as light-absorption enhancers that promote oxidation reactions, contributing to more effective cancer cell eradication [84]. Gu et al. investigated g-C₃N₄-based quantum dot complexes against human oral cancer cells, demonstrating over 83% cancer cell destruction under laser irradiation with ROS production and some oxygen generation, suggesting both therapeutic and drug delivery potential under light-activated conditions in vitro [85]. Yet, variability in tumour redox status across cancer types may influence therapeutic reproducibility.

Collectively, g-C₃N₄-based nanomaterials represent a versatile phototherapeutic platform integrating PDT, PTT, and chemo-/chemodynamic strategies. Despite encouraging preclinical evidence, several translational challenges persist, including limited light penetration depth, insufficient long-term toxicity evaluation, scalability of reproducible synthesis, and incomplete pharmacokinetic profiling. Addressing these limitations through standardized irradiation protocols, comprehensive biosafety studies, and clinically relevant tumour models will be essential before meaningful clinical application can be realized.

**Table 3:** Summary of the most recent applications of g-C_3_N_4_-based nanomaterials in cancer therapy

Composition	Chemotherapeutic drug	Composite preparation	Therapy	Assessment methods	Findings	Refernces
Copper II- g-C_3_N_4_ (Cu^2+^–g-C_3_N_4_)	No additional drug	Cu²⁺-doped g-C_3_N_4_ studied for glutathione depletion to enhance light-triggered ROS production	PDT	TD-DFT, flow cytometry, and MTT assay (HeLa cells)	Enhanced PDT efficacy through Cu²⁺-induced ROS generation and intracellular glutathione reduction	[78]
g-C_3_N_4_- 2-phenylethynesulfonamide (g-C_3_N_4_- PES)	2-Phenylethynesulfonamide (PES; HSP70 inhibitor)	PES-loaded g-C_3_N_4_ nanosheets for HSP70 inhibition to enhance ROS-mediated PDT efficacy	PDT	MTT assay (MCF-7 cells)	HSP70 inactivation enhanced photocontrolled PDT efficacy in cancer cells	[86]
g-C_3_N_4_ − 4-diamino-6-phenyl-1,3,5-triazine and g-C_3_N_4_ -2,4,6-trihydrazino-1,3,5-triazine quantum dots (g-C_3_N_4_- DPT and g-C_3_N_4_- THDT QDs)	No additional drug	Engineered g-C_3_N_4_ QDs with two-photon catalytic capabilities to generate ROS	Two-photon excited PDT (TPE-PDT)	MTT assay (MCF-7 cells)	Improved PDT due to ROS generation	[87]
Thulium/g-C_3_N_4_ /copper phosphide; (Tm/g-C_3_N_4_/Cu_3_P)	No additional drug	Tm/g-C_3_N_4_/Cu_3_P encapsulated in ZIF8 and folic acid-modified for enhanced permeability and receptor-mediated uptake	PTT, PDT, and chemotherapy	MTT assay, fluorescence imaging, in vivo tumour therapy (mice), histology, and Western blot	Elevated temperature in PPT improved chemodynamic therapy by accelerating Cu(I)-H_2_O_2_ fentron reaction	[65]
Chlorin e6@ protonated g-C_3_N_4_ -reduced graphine oxide-polycaprolactone/gelatin (Ce6@pCN-GO-PG)	No additional drug	Ce6@pCN-GO-PG scaffold to improve stability and enhance the sonophotodynamic effect	Sonophotodynamic therapy	NIR and cytotoxicity assays (SKBR3 cells), cytocompatibility with several cells	Efficient inactivation of breast cancer cells by combined PDT and sonodynamic effects	[77]
Zeolitic-imadazolate framework-8/g-C_3_N_4_ (ZIF8/ g-C_3_N_4_/DOX)	Doxorubicin	Doxorubicin-loaded ZIF8/ g-C_3_N_4_ nanosheets for combinatorial PDT-chemotherapy with dual-color imaging	PDT and chemotherapy	MTT assay (A549 cells), EDX, DLS, XRD, and FTIR	Enhanced therapeutic effects via combined chemotherapeutic and PDT effects	[69]
g-C_3_N_4_ quantum dots embedded in carbon nanosheets loaded with Doxorubicin (g-C_3_N_4_QD- CN-DOX)	Doxorubicin	One-pot hydrothermal preparation of DDS enabling controlled drug release, improved light-to-heat conversion, and singlet oxygen generation	PTT, PDT, and chemotherapy	MTT assay (HeLa, NCI-H196, and MCG-803 cells), NIR imaging (mice), and hematology assays	Enhanced cytotoxicity due to NIR-induced local heating and ROS production, strong NIR fluorescence supports imaging-guided cancer therapy	[79]
Cerium Niobate/graphitic carbon nitride (Ce_3_NbO_7_/g-C_3_N_4)_	Cerium Niobate	Ce_3_NbO_7_/g-C_3_N_4_ nanocomposites with high NIR absorption and PTT potential	PTT + CDT	NIR and MTT assay (HepG-2 cells)	Dual therapeutic effect with 68% cancer cell death and 49.5% photothermal conversion efficiency, biocompatible and non-toxic	[76]
g-C_3_N_4_ complex: PGC-Ce6 [PVP-(g-C_3_N_4_ -Cu)-Ce6]	No additional drug	Synthesized using polymer, precursor melamine, metal, and photosensitizer	PDT	MTT assay	Demonstrating over 83% cancer cell destruction against human oral cancer cells, under laser irradiation with ROS production and some oxygen generation	[85]

PTT, photothermal therapy; PDT, photodynamic therapy; TD-DFT, Time dependent density functional theory; CDT, Chemo dynamic therapy; NIR, Near infrared; TPE-PDT, Two photon excited photo dynamic therapy; ROS, Reactive Oxygen Species; HSP70, PES-induced heat shock protein; EDX, Energy dispersive X-ray spectroscopy; DLS, Dynamic light scattering; Ce6, chlorine e6

### Tissue engineering and regeneration

Additionally, g-C₃N₄ has gained considerable attention in regenerative medicine and tissue engineering owing to its excellent biocompatibility, tunable surface chemistry, and ability to support cellular adhesion and proliferation [63]. Its structural versatility allows incorporation into diverse biomaterial systems, including hydrogels [17], nanocomposites [88], and porous scaffolds [89], enabling controlled support for cell proliferation, migration, and differentiation.

Recent studies have highlighted g-C₃N₄-based scaffolds as promising candidates for bone, cartilage, and neural tissue regeneration. For instance, polycaprolactone/g-C₃N₄ (PCL/g-C₃N₄) composite scaffolds demonstrated superior biocompatibility, biodegradability, and mechanical strength compared with pure polycaprolactone, supporting osteogenic differentiation of MC3T3-E1 cells [16]. Similarly, Dutta et al. [90] developed a 3D-printed nanophotocatalytic bactericidal scaffold based on alginate-gelatin/g-C₃N₄ hydrogel that modulated the bone immune microenvironment, promoting anti-inflammatory and osteo-immunomodulatory responses for enhanced bone repair [90].

Beyond bone regeneration, g-C₃N₄-functionalized scaffolds have been shown to support fibroblast, mesenchymal stem cell, and osteoblast growth, as well as to accelerate wound healing by promoting angiogenesis, tissue repair, and re-epithelialization [89]. Additionally, polycaprolactone micropatterns functionalized with g-C₃N₄ have been explored for neural tissue engineering, demonstrating enhanced neural stimulation and electrical conductivity when tested with rat adrenal cells, suggesting their potential as nerve guidance conduits for peripheral nerve repair [91]. Recent studies have begun to explore g-C₃N₄ nanocomposites for regenerative dental applications. For example, a CeO₂-g-C₃N₄-BA nanoparticle system demonstrated potent antibacterial activity and significantly enhanced alveolar bone regeneration in periodontitis animal models, mediated via antioxidant and osteogenic mechanisms, highlighting dual functionality relevant for periodontal wound healing [92]. Comprehensive reviews also identify such composites as promising multifunctional agents that integrate antimicrobial and regenerative properties for next-generation dental biomaterials, although clinical translation remains in its early stages [93].

While g-C₃N₄ and its derivatives show promising performance in tissue engineering and regenerative medicine, their biodegradation behaviour and in vivo resorption remain important considerations for long-term biomedical safety and scaffold performance. Swetha et al. demonstrated that both exfoliated and porous g-C₃N₄ nanosheets are susceptible to enzymatic degradation by human myeloperoxidase and horseradish peroxidase, with porous structures showing enhanced degradability due to surface oxygen functionalities, indicating the potential for biological breakdown under oxidative conditions [94]. Additionally, in a rabbit femoral defect model, a g-C₃N₄/graphene oxide composite scaffold exhibited partial in vivo resorption over time alongside enhanced bone regeneration, suggesting that g-C₃N₄–based composites can undergo degradation and integration within physiological environments [95]. These studies underscore the multifaceted potential of g-C₃N₄-based biomaterials in promoting cellular regeneration through their photocatalytic activity, surface reactivity, biodegradibility, and biofunctionality, offering promising prospects for next-generation scaffolds in regenerative medicine.

### Wound healing

The ability of g-C_3_N_4_ to modulate multiple tissue repair pathways, along with its inherent antibacterial properties, makes it an attractive material for wound healing applications. A summary of the most recent studies is presented in Table 4. Various formulations, such as membrane hydrogels, nanocomposites, and macropatches incorporating g-C_3_N_4_ with antibacterial agents, have exhibited enhanced wound healing activity [9, 10]. Furthermore, recent studies confirmed the high biocompatibility and clinical safety of g-C_3_N_4_-based wound dressing, highlighting their therapeutic potential in the management of chronic and diabetic wounds [71, 96]. Notably, silver-based g-C_3_N_4_ hydrogels (AgNPs-g-C_3_N_4_/PVA) have demonstrated excellent antibacterial activity against Escherichia coli (*E. coli*) while maintaining strong mechanical properties [97]. Additionally, its clinical efficacy against drug-resistant bacterial infections has also been reported, further highlighting its versatility and promise as next-generation wound-healing biomaterials [98].

Recent studies have highlighted the importance of modulating the ionic microenvironment to enhance the photodegradation efficiency of g-C_3_N_4_, thereby accelerating the wound healing process [99]. The formation of an optimized ionic microenvironment enhances thermal exfoliation and light-driven catalytic activity, leading to faster tissue repair through improved degradation of cellular debris and reactive species. Further, the antibacterial effectiveness of g-C_3_N_4_-based composites has been demonstrated against Methicillin-Resistant Staphylococcus Aureus (MRSA) using a Zinc-doped g-C_3_N_4_ nanocomposite, supporting its role in treating wounds infected with drug-resistant bacteria [15, 71]. In addition, g-C_3_N_4_-based polylactic acid (PLA) hydrogel bandages have shown the ability to generate oxygen under NIR, creating an anti-inflammatory microenvironment that promotes wound healing and tissue regeneration [100].

Rapid wound healing has also been reported using manganese oxide-based g-C_3_N_4_ nanocomposites, which effectively reduced the bacterial biofilm formed by *Staphylococcus aureus (S.aureus)* and *Pseudomonas aeruginosa* (*P.aeruginosa*) in a rat skin injury model [101]. In another in vivo study, Liu et al. reported faster wound healing in a mouse model treated with a photoactive, self-healing macropatch composed of g-C_3_N_4_ and carboxymethyl chitosan, emphasizing the patch’s excellent adhesion and faster wound healing properties [17]. The macropatch showed a significant hemostatic effect on liver wounds, achieving a 67.7% reduction in bleeding compared to untreated mice. Additionally, g-C_3_N_4_ nanocomposites combined with natural plant extracts (e.g., eucalyptus) have shown strong bactericidal effects against *S. aureus* and *E. coli*, along with enhanced fibroblast proliferation and cell migration demonstrated by NIH-3T3 scratch assays [88].

Although g-C₃N₄–based materials have shown promising wound-healing effects in general soft-tissue models through antibacterial and ROS-mediated mechanisms, oral and maxillofacial wounds present unique challenges, including persistent saliva exposure, polymicrobial biofilms, fluctuating pH, and the close coupling of soft-tissue healing with alveolar bone regeneration. Consequently, recent studies have begun to explore g-C₃N₄ in dental and periodontal wound-healing settings and can effectively reduce oral bacterial burden under visible light, thereby limiting infection-driven inflammation in periodontal wound environments [93]. One study investigated g-C₃N₄-containing nanocomposites designed for periodontal wound environments, where chronic bacterial infection significantly impairs healing [102]. The latter study incorporated g-C₃N₄ as a visible-light–activated filler in dental resin composites, achieving > 99% kill rate of *Streptococcus mutans* (*S. mutans*) and *S. aureus* while retaining mechanical performance and biocompatibility, with direct relevant to dental restorative applications and bacterial control [102]. Overall, current evidence suggests that g-C₃N₄ supports dental wound healing primarily through infection control, inflammation reduction, and redox regulation, though further studies under saliva-mimicking and clinically relevant oral conditions are still needed. Another study focused on a g-C₃N₄-based heterostructure nanomaterial evaluated in a periodontitis-associated defect model, which closely mimics oral wound healing involving both soft tissue and alveolar bone [92]. Beyond antibacterial activity, the composite promoted osteogenic differentiation and reduced oxidative stress in periodontal tissues. The dual regulation of ROS and inflammation contributed to improved tissue regeneration at defect sites [92]. Additionally, photocatalytic g-C₃N₄ systems designed for dental applications have been reported to disrupt oral biofilms while maintaining cytocompatibility with oral cells, leading to improved wound-closure responses under bacterial challenges in vitro [93].

These findings highlight the therapeutic versatility of g-C₃N₄-based materials in wound management, with emerging potential for oral applications, especially when combined with antibacterial agents to address drug-resistant infections and promote healing in chronic or diabetic wounds.

**Table 4:** Summary of animal studies in wound healing applications using g-C₃N₄-based nanomaterial preparations

Composition	Preparation methods	Type of preparation	Experimental models (duration)	Microorganism	Findings	References
Carboxymethyl chitosan/tannic acid/g-C_3_N_4_ (CMCS/TA/g-C_3_N_4_)	Integrative one-step approach	Hydrogel macropatch	Mouse skin and rat liver wounds (14 days)	*S.aureus* and *P.aeruginosa*	Excellent hemostatic effect on liver bleeding, improved antibacterial properties, and accelerated skin wound healing	[17]
Calcium-carbon-loaded silver-based carbon dot doped g-C_3_N_4_ and chitosan CaCO_3_@AgCCN/CS)	Doping and integration	Dual-responsive pH/red light nanocomposite hydrogel	Mouse skin wound treatment (14 days)	*S.aureus* and *E. coli*	Controlled CO delivery by AgCCN and the red-light illumination improved antibacterial and anti-inflammatory effects	[9]
Poly vinyl alcohol/Bacterial cellulose/Calcium/ g-C_3_N_4_/Aloe vera (PVA/BC/g-C_3_N_4_/Calcium/Aloe Vera)	Thermal treatment and component integration	Nanofibers	Mouse skin wound treatment (21 days)	*G. xylinus*, *S.aureus*, and *E. coli*	Accelerated healing of diabetic lesions and robust mechanical properties	[103]
g-C_3_N_4_/manganese oxide/poly-L-lactic acid (g-C_3_N_4/_MnO_2_/PLLA)	Electrospinning	Nanosheets	Rat-skin injury model (14 days)	*S.aureus* and *P.aeruginosa*	83 and 62% biofilm elimination, and accelerated wound healing by pro-angiogenic regeneration and tissue remodeling	[101]
g-C_3_N_4_-based silk cocoons (g-C_3_N_4_/FSCs)	Grafting and integration	Membrane wound dressings	Antibacterial assays, Rat skin wound treatment (8 days)	*S. aureus* and *E. coli*	High bactericidal efficiency (99.9%) and fast infected wound healing with increased photoactivity and biocompatibility	[104]
Bacterial cellulose/Poly-vinyl alcohol/g-C_3_N_4_/Trachyspermum (PVA/BC/ g-C_3_N_4/_Nettles/ trachyspermum)	Electrospinning	Nanocomposite wound dressing	Cytotoxicity, migration, proliferation, antibacterial assays, and mouse-skin wound treatment (14 days)	*S. aureus* and *E. coli*	Non-toxic composite accelerates the wound healing process to 95% with excellent antibacterial property	[105]
Silver nanoparticles g-C_3_N_4_ /poly-vinyl-acid (AgNPs/g-C_3_N_4_ /PVA)	Cyclic freeze-thaw	Bactericidal hydrogel	Mouse skin wound treatment	*E. coli*	Good bactericidal activity and enhanced mechanical properties	[97]
Zinc hexacyanoferrate g-C_3_N_4_ (Zn_3_[Fe (CN)_6_]/g-C_3_N_4_)	In situ loading of nanospheres onto g-C_3_N_4_ sheets	Nanocomposite	Mouse skin wound treatment (10 days)	*S. aureus* and *E. coli*	High bactericidal efficacy and 99.22% wound closure within 10 days	[71]
Zn-doped g-C_3_N_4_ phosphorous-doped graphene oxide/ bismuth sulfide (Zn-doped/g-C_3_N_4_/P-GO/Bi_2_S_3_)	Chemical vapor deposition and doping	Heterostructure	Cytotoxicity, scratch assays, rat skin wound treatment (12 days)	MRSA	99.6% MRSA elimination and enhanced wound healing.	[98]
Poly lipoic acid/tromethanol/ dopamine/ g-C_3_N_4_ (PLA/Ce_3+_/ DA/g-C_3_N_4_)	Injection molding with NIR photothermal enhancement	Injectable hydrogel bandage	Lap-shear, biocompatibility, and mouse skin wound treatment (14 days)	-	Wound healing is promoted by oxygen generation and an anti-inflammatory microenvirnment	[100]
Ca-doped/g-C_3_N_4_/ manganese sulfide (Ca-doped/g-C_3_N_4_/ MnS)	Two-photon excitation and doping	Heterojunction nanocomposite		-	Effective bactericidal action triggering cellular metabolism and tissue repair	[15]
g-C_3_N_4_ modified by ionic liquids (g-C_3_N_4_-ILs)	Ionic liquid microenvironment modulation via non-covalent interactions	Liquid formulation	Cytotoxicity, scratch assays, mouse skin wound treatment (8 days)	*S. aureus*	Enhanced photodegradation efficiency and bacterial wound healing, improved collagen synthesis and re-epithelialization	[99]
g-C_3_N_4_ / bisphenol A glycidyl methacrylate/triethylene glycol dimethacrylate (g-C_3_N_4 −_Bis-GMA/TEGDMA)	One-step thermal polycondensation and doping	Visible-light–activated filler in dental resin compounds	Cytotoxicity, scratch assays	*S. aureus*	> 99% kill rate of *Streptococcus mutans* and *S. aureus* while retaining mechanical performance and biocompatibility	[102]

S. aureus, Staphylococcus aureus; P. aeruginosa, Pseudomonas aeruginosa; E. coli, Escherichia coli; G. xylinus, Gluconacetobacter xylinus; MRSA, Methicillin resistant Staphylococcus aureus; NIR, Near infrared

### Biosensing and bioimaging

Various forms of g-C₃N₄ have recently attracted significant attention for bioanalytical and biomedical applications, with a summary of recent studies listed in Table 5. The unique photoelectrochemistry, photoluminescence, electrochemiluminescence, and fluorescence properties of g-C₃N₄ provide a broad platform for its use in biosensing and bioimaging [19–22]. Its structural characteristics, such as a large surface area, tunable electronic configuration, and the ability to engage in hydrophobic and electrostatic interactions, further enhance its competence in diagnostic applications and analytical systems [106]. Biosensors incorporating g-C₃N₄-based materials have been successfully developed for detecting various biomolecules, including nucleic acids, proteins, membrane biomarkers (particularly cancer markers), and pathogens, including viruses and bacteria [80, 107, 108], carbohydrates (e.g., glucose), and enzymes such as alkaline phosphatase in biological fluids [19, 109, 110]. Based on the current literature, g-C₃N₄-based biosensing systems can be broadly categorized into four main types: (1) surface plasmon resonance biosensors, (2) photoelectrochemical and electrochemical biosensors, (3) fluorescence biosensors, and (4) electro-chemiluminescent biosensors.

Surface plasmon resonance biosensors operate based on the physical optics phenomenon of surface plasmon resonance, providing high-sensitivity detection of refractive index changes at the sensor surface within a dielectric medium [111]. Due to g-C₃N₄ strong light-responsive properties, surface plasmon resonance biosensors incorporating this material exhibit remarkable accuracy and sensitivity in detecting single cells’ antigen-antibodies, proteins, and anticancer drugs [112–114]. On the other hand, the ability of g-C₃N₄ to generate or transfer electrons through enzymatic catalytic reactions, along with its excellent coupling compatibility with other nanomaterials, makes it an ideal candidate for photoelectrochemical and electrochemical biosensors with high sensitivity and accuracy [23, 115, 116]. The derivatives of g-C₃N₄ can electronically interact with biomolecular redox centers, which can be detected by specialized sensors [117]. Recent advances in electrochemical biosensors based on g-C₃N₄ have demonstrated high specificity and sensitivity in detecting circulating cancer cells in brain cancer (glioma) [118]. Additionally, the photoelectrochemical biosensors that convert photoirradiation into measurable electrical signals are prominent due to their high capacity and sensitivity in detecting clinically relevant biomolecules, including glucose, microRNA, methylated RNA, dopamine, and carcinoembryonic antigen in human serum samples [83, 116, 119, 120]. Fluorescence biosensors that target cells with high specificity are extensively used in biomedical diagnostics, particularly for cancer detection [25]. These biosensors operate by absorbing electromagnetic radiation through fluorophores conjugated to fluorescently labelled molecules, resulting in emitting detectable fluorescence signals [121]. Studies have demonstrated that surface modifications of g-C₃N₄ can enhance its fluorescence quantum yield, significantly improving its sensitivity for biomolecule detection [107, 122]. In parallel, electro-chemiluminescent biosensors work by detecting analytes, such as nucleic acids, peptides, and enzymes, by producing luminescent signals whose intensity correlates with the analyte concentration [24]. Recently, Tian et al. reported the successful development of a g-C₃N₄-based electro-chemiluminescent biosensor coupled with an aptamer for the sensitive detection of aflatoxin B1, demonstrating excellent analytical performance [123]. Similarly, silver nanoparticle-doped g-C₃N₄ nanocomposites have been shown to enhance ECL biosensors, enabling highly accurate detection of neurotransmitters such as dopamine [124]. Furthermore, gold-fabricated g-C₃N₄ nanoparticles exhibited a wide detection range from 10 fg/mL to 50 ng/mL to detect amyloid-b proteins in human serum samples [125]. These advancements underscore the remarkable versatility and analytical precision of g-C₃N₄-based biosensors, paving the way for their use in the ultrasensitive detection of trace biomolecules and clinically relevant targets in medical diagnostics. However, it should be noted that the majority of g-C₃N₄-based biosensing platforms reported to date remain at the proof-of-concept stage, with performance largely evaluated under laboratory conditions, and further validation using clinically relevant samples is required before translational applications can be realized.

**Table 5:** Summary of the most recent applications of g-C₃N₄-based nanomaterials as biosensors

Sensors	Materials detected	Analytical device	Findings	References
*Surface plasmon resonance (SPR) biosensors*				
g-C_3_N_4_/chitosan (g-C_3_N_4_/CS)	Flutamide (anticancer drug)	Nanocomposites sensor	Demonstrated a linear response from 1 to 150 µM, achieving a detection limit of 120 nM with its high sensitivity	[114]
Gold and chitosan embedded g-C_3_N_4_ (Au/g- C_3_N_4_/CS)	C-reactive protein	Nanofilm-based surface plasmon resonance biosensor	Detection of a diagnostically relevant range of the biomarker in serum. achieved a LOD of 2.2 ng/mL for C-reactive protein in spiked serum samples without the need for signal amplification	[126]
*Photoelectrochemical (PEC) biosensors*				
Carboxylated g-C_3_N_4_/Titanium dioxide (C-g-C_3_N_4_/TiO2)	Carcinoembryonic antigen	Nanosheet photoelectrochemical biosensor	Excellent photocatalytic activity with specificity for the detection of carcinoembryonic antigen in human serum samples (with a detection concentration in the range of 0.01–10 ng/mL, to 2.1 pg/mL)	[116]
ND- g-C_3_N_4_	MicroRNA from cancer cells	Nanosheet photoelectrochemical biosensor	Accurate detection of microRNA from cancer cells with a convertible target microRNA strategy (LOD = 0.8 fM (S/*N* = 3)	[120]
*Electrochemical biosensors*				
Probe DNA/oxidized g-C_3_N_4_ nanosheets (PDNA/Ox/g-C_3_N_4_)	Viral DNA (norovirus)	Electrochemical paper-based analytical device	Cost-effective, portable, and disposable sensor with high selectivity and sensitivity towards norovirus DNA	[23]
g-C_3_N_4_/nonporous gold (g- C_3_N_4_ /NPG)	Circulating glioma cells (Brain cancer)	Nanocomposites electrochemical sensor	Displayed high sensitivity, low detection limit, and strong anti-interference ability for detecting circulating cancer cells in glioma human serum samples (linear detection range from 1 to 1 × 10⁶ cells/mL, with a minimal detection limit of 1 cell/mL)	[118]
*Electrochemiluminescent (ECL) biosensors*				
Silver-doped g-C_3_N_4_ (g- C_3_N_4_@Ag)	Dopamine	Electrochemiluminescent probe	The lowest detection limit of 44 mM dopamine was achieved using a metal nanoparticle modification strategy	[124]
g-C_3_N_4_ nanosheets fabricated with gold nanoparticles (g-C_3_N_4_@Au NPs)	Amyloid-protein	Metal–organic framework-based Electrochemiluminescen sensor	A wide detection range of 10 fg/mL to 50 ng/mL with high specificity for amyloid-β detection in human serum samples	[125]
*Fluorescence biosensors*				
g-C_3_N_4_ embedded with hyaluronic acid (g-C_3_N_4_@HA)	Cancer cell imaging	Microgel fluorescence sensor	Demonstrated strong cell-targeting and penetration capability linked to hyaluronic acid mediated binding to cancer cell receptors	[107]
Folic acid and sulfur-doped g-C_3_N_4_ (FA/S, O/g-C_3_N_4_)	Folic acid	Nanocomposite fluorescence probe	Highly sensitive and selective folic acid detection in the range 5.0–83.3 µM with a detection limit of 90 nM	[108]

_LOD = Limit of detection_

Taken together, these advancements open new avenues for g-C₃N₄ based biosensors to be applied in a wider range of biomedical applications. The continued evolution of these technologies holds great promise for the development of highly sensitive and selective diagnostic platforms, enabling earlier disease detection, more accurate clinical assessments, and ultimately improved therapeutic outcomes and patient care.

## Challenges and future perspectives

### Fundamental material and biological hurdles

Despite the substantial promise of g-C₃N₄ in various biomedical applications, several fundamental challenges must be addressed. A primary hurdle lies in production, while g-C₃N₄ can be synthesized through relatively simple methods, achieving large-scale production with consistent quality, properties, and uniform morphology remains a significant barrier [41, 127]. The development of scalable and reproducible synthetic routes is essential for ensuring consistent research outcomes and for the eventual widespread use of g-C₃N₄ in regulated biomedical products.

Beyond synthesis, the intrinsic properties of g-C₃N₄ require optimization. Its photocatalytic efficiency, for instance, is often limited by a high rate of electron-hole recombination and a narrow light absorption window [128, 129]. However, several engineered strategies have demonstrated improved performance in reported antibacterial systems. For instance, heterojunction formation (e.g., g-C₃N₄/CdS-Cu or Bi-based composites) enhances charge separation efficiency, resulting in antibacterial rates exceeding 95% against *E. coli* and *S. aureus* under visible light irradiation, primarily due to increased ROS generation [130]. Similarly, CuS@g-C₃N₄ heterojunction systems have shown enhanced biofilm eradication and antibacterial activity, attributed to reduced electron–hole recombination and synergistic photodynamic effects [131]. Strategies to enhance its performance, such as doping, creating heterojunctions, or incorporating co-catalysts, are crucial for maximizing therapeutic efficacy [41, 132]. Similarly, the poor dispersibility of g-C₃N₄ sheets in aqueous media due to strong van der Waals forces reduces its effective surface area and limits its utility [133, 134]. While chemical modifications with hydrophilic polymers can improve this, the complexity and cost of these functionalization processes can impede scalability [134]. Although multimodal therapeutic platforms frequently report enhanced efficacy, the precise contribution of each modality is often not systematically isolated, and mechanistic interpretations are commonly based on indirect evidence. Developing more efficient, cost-effective, and facile modification strategies therefore remains a critical ongoing challenge. Future studies should incorporate well-designed control experiments and quantitative analytical approaches to delineate the individual and synergistic contributions of each therapeutic component, enabling more robust mechanistic validation and rational system optimization.

Biomedical safety considerations for nanomaterials remain insufficiently addressed. Multiple recent studies emphasize that ion leaching (e.g., release of Cu²⁺, Ce³⁺, or other dopant species), surface reactivity–induced oxidative stress, and dose-dependent cytotoxicity can significantly influence cellular viability and inflammatory responses [135–138]. Moreover, long-term biodistribution, degradation kinetics, and chronic biological effects remain underexplored, particularly for hybrid and doped nanoparticle systems [139–141]. These highlight the need for a systematic evaluation of ion release behavior, cytocompatibility thresholds, and long-term in vivo safety before clinical translation of doped nanomaterials.

The current understanding of g-C₃N₄ biocompatibility is incomplete and relies predominantly on short-term in vitro assays. While these studies provide important preliminary safety information, they fail to capture long-term biological responses, including chronic inflammation, immune modulation, or organ accumulation [7, 142]. This assessment is further complicated by the use of composite formulations rather than pristine g-C₃N₄ alone. Several g-C₃N₄-based systems incorporate components, such as metal oxides or nanoparticles that possess known dose-dependent cytotoxicity [18, 74]. It is essential to distinguish the intrinsic biocompatibility of the g-C₃N₄ platform from the biological effects of these additives. Therefore, comprehensive in vivo toxicity assessments must be interpreted in a component-resolved manner.

In addition to toxicity considerations, the biodegradation behaviour of g-C₃N₄ in vivo remains an important but incompletely resolved aspect of its safety profile. Due to its polymeric and chemically stable framework, pristine g-C₃N₄ is not expected to undergo rapid enzymatic degradation under physiological conditions [7]. However, experimental evidence indicates that g-C₃N₄ can undergo slow structural fragmentation through oxidative or photo-induced pathways, particularly when engineered at the nanoscale or incorporated into defect-rich or composite systems [35]. Such processes are proposed to yield low-molecular-weight carbon- and nitrogen-containing species, although direct in vivo identification of degradation by-products and clearance routes remains limited. Consequently, comprehensive long-term in vivo studies are required to elucidate degradation kinetics, metabolite fate, and organ clearance, representing a critical requirement for future clinical translation. While oxidative biodegradation has been suggested, the nature of the resulting degradation by-products and their toxicity profiles remains incompletely understood and warrants further investigation.

### Research priorities for dental translation

**Fig. 6:**
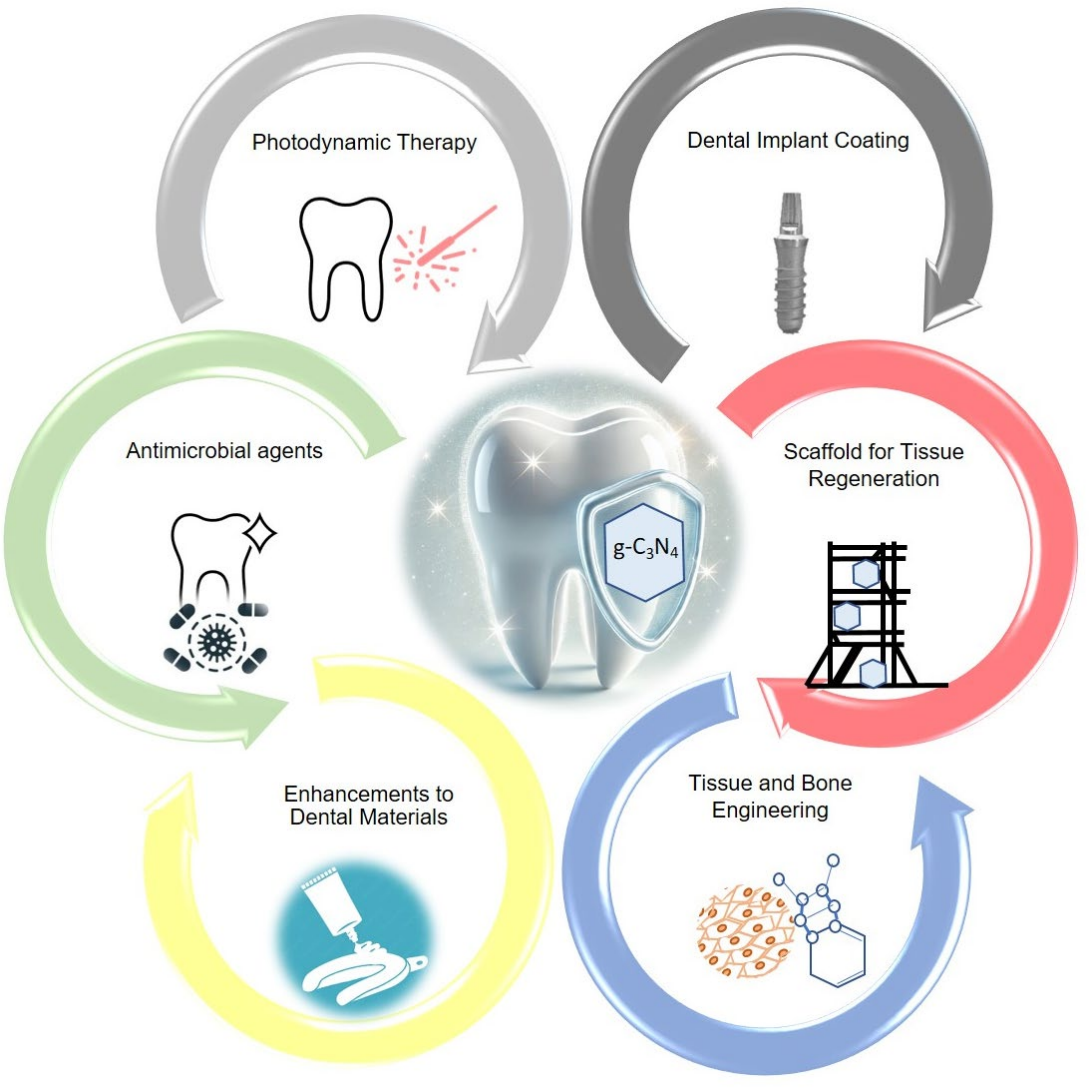
Schematic illustration of potentional dental applications of g-C_3_N_4_-based nanomaterials

**Fig. 7:**
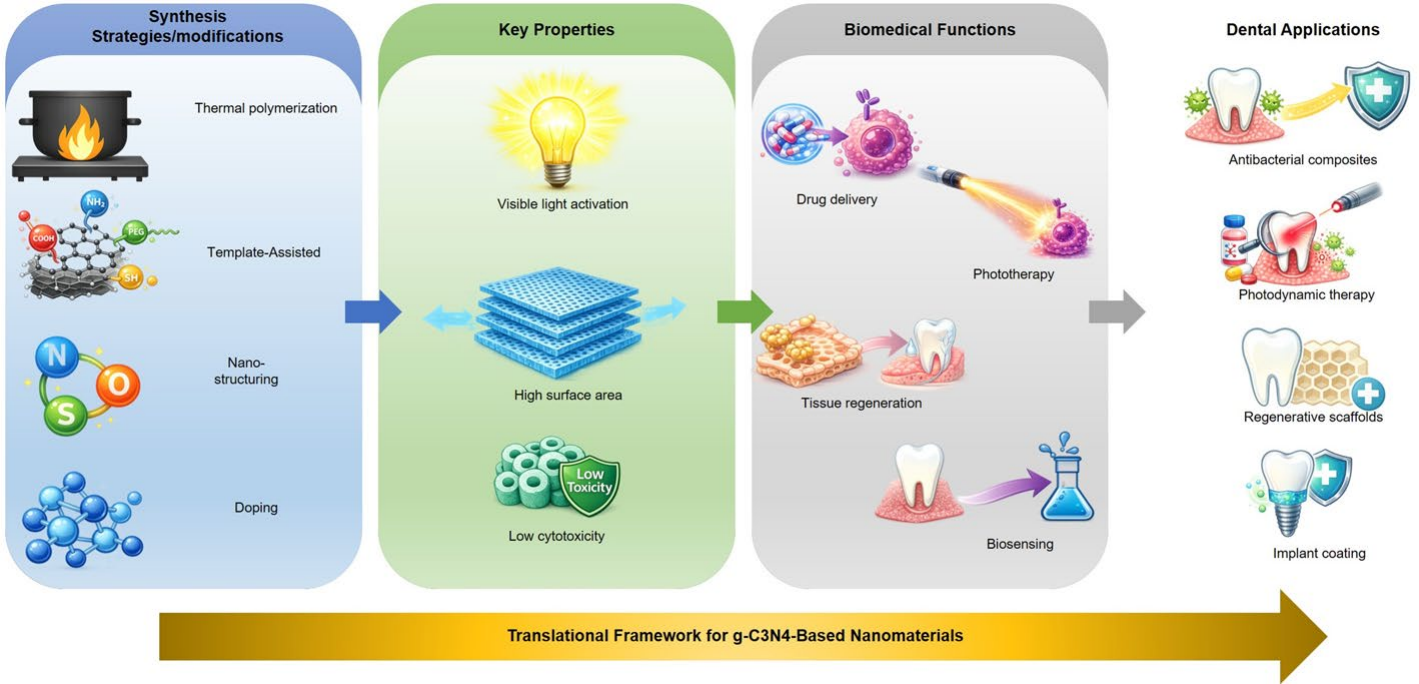
Conceptual summary of the translational framework for g-C₃N₄-based nanomaterials. This graphic illustrates how specific synthesis strategies modulate key properties (e.g., visible light activation, high surface area). These properties, in turn, enable versatile biomedical functions (e.g., drug delivery, tissue regeneration), which translate into advanced dental applications such as antibacterial composites, regenerative scaffolds, and implant coatings

Translating g-C₃N₄-based systems into clinical dentistry requires addressing the uniquely dynamic nature of the oral cavity. Any candidate material must be specifically engineered to overcome a combination of demanding environmental factors that dictate its long-term stability and efficacy. These include: (i) the constant diluting and clearing effect of salivary flow; (ii) dynamic and often acidic pH fluctuations driven by diet and bacterial metabolism; (iii) the persistent formation of dense, multi-species biofilms [143]; and (iv) significant and repetitive mechanical forces from mastication. As illustrated in Fig. 6 and 7, the established biomedical properties of g-C₃N₄ directly map onto key clinical challenges in dentistry, from inhibiting biofilms to promoting tissue regeneration. However, to bridge the gap from potential to practice, and as detailed in Table 6, future research must be explicitly designed to test materials against these challenges.

**Table 6:** Translational relevance and clinical application mapping of g-C₃N₄ properties in dentistry

Properties of g-C₃*N*₄	Level of biomedical evidence	Target dental clinical challenge	Potential dental application
Intrinsic antimicrobial and antibiofilm activity	Effective against gram-positive, gram-negative, fungal, and drug-resistant pathogens; ROS-mediated bacterial killing [144]	Dental plaque biofilm, secondary caries, peri-implantitis, endodontic infections	Antimicrobial coatings for dental implants, orthodontic brackets, endodontic files, restorative fillers
Photodynamic and photothermal responsiveness	Light-triggered ROS generation and localized hyperthermia for cancer therapy and wound disinfection [145]	Periodontal infections, deep carious lesions, oral precancerous lesions, adjunctive periodontal therapy	Light-activated dental gels, fiber-optic-assisted photodynamic and photothermal systems, antimicrobial phototherapy adjuncts
Drug loading and controlled release capability	pH- and light-responsive delivery of chemotherapeutics [13], antibiotics, and bioactive agents [146]	Local drug delivery in periodontal pockets, pulp therapy, post-surgical infection control	Drug-eluting dental composites, sealers, hydrogels, and periodontal delivery systems
Biocompatibility and support of cell adhesion and proliferation	Supports the proliferation of various cell types (e.g., fibroblasts and osteoblasts) for tissue and bone regeneration [89]	Pulp–dentin complex repair, periodontal regeneration, bone healing around implants	g-C₃N₄-functionalized membranes and scaffolds for guided tissue/bone regeneration
Oxygen generation and microenvironment modulation	Alleviates hypoxia, reduces inflammation, and enhances wound healing [17]	Chronic periodontal inflammation, impaired healing in diabetic or compromized patients	Oxygen-generating wound dressings, regenerative periodontal scaffolds
Fluorescence and photoelectrochemical properties	Enables high-sensitivity biosensing of biomarkers and pathogens, alongside applications in fluorescent cellular bioimaging [22, 107]	Early caries detection, periodontal diagnostics, oral cancer screening	Chairside biosensors, fluorescence-based diagnostic probes, bioimaging tools
Tunable surface chemistry and composite compatibility	Easy integration with polymers, metals, and bioceramics [63, 80]	Mechanical reinforcement and antimicrobial enhancement of dental materials	Bioactive restorative materials, adhesive systems, implant surface modifications

ROS, reactive oxygen species

### The key research priorities should therefore include

*Development of Wear-Resistant, Drug-Eluting Antimicrobial Composites:* A primary opportunity lies in leveraging g-C₃N₄ antimicrobial and drug-eluting capabilities to combat biofilm-driven infections responsible for secondary caries and peri-implantitis. Future studies must focus on incorporating g-C₃N₄ as a functional filler into dental resin composites, cements, or adhesives. A critical short-term milestone is to optimize filler concentration and surface chemistry to ensure strong covalent bonding within the resin matrix, preventing nanoparticle leaching while maximizing antimicrobial efficacy against key oral pathogens. In the medium-term, these resin composites must be subjected to rigorous mechanical wear and long-term aging studies in simulated oral environments to validate that their durability is on par with current gold-standard dental materials.

*Validation of Phototherapy for Endodontic and Periodontal Disease:* The photoresponsive properties of g-C₃N₄ make it an attractive agent for photodynamic and photothermal therapies against deep carious lesions and periodontal infections. However, research must advance beyond static in vitro assays toward dynamic, multi-species biofilm models grown on extracted human teeth. A key challenge is to design effective light-delivery strategies, such as fiber-optic systems, to ensure the required light energy can penetrate complex anatomical sites like root canals or deep periodontal pockets and activate the material.

*Design and Validation of Bio-interactive Regenerative Scaffolds:* For applications in guided bone or tissue regeneration around implants or in periodontal defects, g-C₃N₄ ability to support cell proliferation is highly relevant. Future research should focus on creating bio-interactive scaffolds or membranes that leverage this property. Studies must systematically investigate how different g-C₃N₄ surface modifications influence the adhesion, proliferation, and differentiation of osteoblasts and periodontal ligament stem cells. The long-term goal is to develop scaffolds that not only provide antimicrobial protection to the healing site but also actively guide and promote the desired regenerative response.

### Pathway to the clinic: a realistic outlook

A realistic assessment of g-C₃N₄ translational potential must involve quantitative benchmarking against established nanomaterials such as titanium dioxide (TiO₂) [147] and graphene oxide (GO) [148]. While TiO₂ exhibits high photocatalytic efficiency under UV irradiation and GO offers exceptional electrical conductivity, both materials present limitations, including UV-restricted activation and dose-dependent cytotoxicity [149, 150]. In contrast, g-C₃N₄ demonstrates visible-light activity and favorable biocompatibility profiles, albeit with a comparatively lower baseline photocatalytic efficiency [151]. Establishing standardized benchmarking metrics for photocatalytic activity, ROS yield, antimicrobial efficacy, and cytocompatibility is a critical first step for objective cross-material evaluation and for guiding rational material selection.

Beyond comparative performance, the path to clinical translation is governed by a complex and stringent regulatory landscape that poses a significant hurdle for all novel nanomaterials [152, 153]. For g-C₃N₄, this challenge is multifaceted. Firstly, there is the crucial issue of classification: a g-C₃N₄-based product could be regulated as a medical device (e.g., an implant coating), a drug (e.g., an injectable phototherapy agent), or a combination product, each with a vastly different and costly approval pathway through regulatory bodies such as the U.S. Food and Drug Administration (FDA) or European Medicines Agency (EMA). Secondly, there is currently no specific regulatory guidance for carbon nitride materials. This forces developers to rely on precedent from other carbon-based nanomaterials (e.g., graphene, nanotubes), creating uncertainty.

Crucially, any submission for clinical trial approval will depend on demonstrating robust Chemistry, Manufacturing, and Controls (CMC) [30]. This requires moving beyond laboratory-scale synthesis to establish a validated, scalable manufacturing process that yields g-C₃N₄ with consistent physicochemical properties including size, charge, purity, and defect density from batch to batch [154]. The lack of such standardization, as highlighted in Sect. 6.1, is currently one of the greatest barriers to initiating the formal regulatory process. To provide a more structured evaluation of translational readiness, the development of g-C₃N₄-based systems can be contextualized within a Technology Readiness Level (TRL)-informed framework, linking the level of experimental validation to stages of clinical translation. Considering these profound scientific and regulatory challenges, the current state of g-C₃N₄ for most dental and biomedical applications resides at a low TRL of 2–4, corresponding to early-stage development predominantly supported by in vitro studies and limited proof-of-concept validation [155, 156]. The journey from this stage through pre-clinical toxicology, GMP manufacturing, and multi-phase human clinical trials is both lengthy and complex.

Progression to higher TRL stages (TRL 5–7) will require robust in vivo validation, reproducible large-scale synthesis, and alignment with regulatory benchmarks including CMC and safety profiling, while advancement to clinical implementation (TRL 8–9) remains contingent on well-designed human trials and long-term outcome data. Consequently, a realistic timeline for the first clinical translation of a g-C₃N₄-based dental product extends well beyond the next decade. Success is contingent not only on solving the fundamental material and biological challenges, but also on proactively engaging with the complex realities of the regulatory pathway. These considerations underscore that the future of g-C₃N₄ in dentistry will depend less on incremental improvements in photocatalytic performance and more on rigorous standardization, safety validation, and regulatory alignment. Framing these challenges within a TRL-informed continuum provides a more transparent and semi-quantitative perspective on the gap between laboratory innovation and clinical application. The field must therefore transit from proof-of-concept demonstrations toward translationally designed studies that anticipate real-world clinical constraints from the outset.

## Conclusion

Although g-C₃N₄ has attracted increasing attention as a potential biomedical material, its path toward clinical translation remains at an early stage. While its high surface area and tunable photo-responsiveness offer clear opportunities for applications such as drug delivery and photodynamic therapy, these attributes alone are insufficient to ensure translational success. A key challenge lies in the lack of standardized and scalable manufacturing productions, which contributes to variability in biological performance, incomplete understanding of bio–nano interactions, and uncertain stability in complex physiological environments. In addition, most biocompatibility assessments rely on short-term in vitro studies, underscoring the need for systematic long-term in vivo investigations addressing toxicity, biodistribution, and clearance. For dental applications in particular, current evidence is limited, with notable gaps in data regarding cytotoxicity under oral conditions, sustained antibacterial efficacy, and long-term resistance to biofilm formation. Addressing these challenges through application driven composite design and rigorous biological validation will be essential for advancing g-C₃N₄ toward meaningful clinical relevance in personalized medicine and dentistry.

## Data Availability

No datasets were generated or analysed during the current study.
